# Mineralization in a Critical Size Bone-Gap in Sheep Tibia Improved by a Chitosan-Calcium Phosphate-Based Composite as Compared to Predicate Device

**DOI:** 10.3390/ma15030838

**Published:** 2022-01-22

**Authors:** Gissur Örlygsson, Elín H. Laxdal, Sigurbergur Kárason, Atli Dagbjartsson, Eggert Gunnarsson, Chuen-How Ng, Jón M. Einarsson, Jóhannes Gíslason, Halldór Jónsson

**Affiliations:** 1 IceTec, 112 Reykjavík, Iceland; 2 Faculty of Medicine, University of Iceland, 101 Reykjavík, Iceland; artvita2@gmail.com (E.H.L.); skarason@landspitali.is (S.K.); ingibjorgsim@internet.is (A.D.); halldor@landspitali.is (H.J.J.); 3 Department of Vascular Surgery, Landspítali University Hospital, 108 Reykjavík, Iceland; 4 Department of Anaesthesia and Intensive Care, Landspítali University Hospital, 108 Reykjavík, Iceland; 5 Institute for Experimental Pathology, University of Iceland, 112 Reykjavík, Iceland; eggun@hi.is; 6 Genis hf., 580 Siglufjörður, Iceland; ngchw@genis.is (C.-H.N.); jon.einarsson@genis.is (J.M.E.); johgisla@gmail.com (J.G.); 7 Department of Orthopaedic Surgery, Landspítali University Hospital, 108 Reykjavík, Iceland

**Keywords:** bone implant, bone defects, chitosan, degree of deacetylation, bone formation, X-ray micro CT, histology, sheep tibia

## Abstract

Deacetylated chitin derivatives have been widely studied for tissue engineering purposes. This study aimed to compare the efficacy of an injectable product containing a 50% deacetylated chitin derivative (BoneReg-Inject™) and an existing product (chronOS Inject^®^) serving as a predicate device. A sheep model with a critical size drill hole in the tibial plateau was used. Holes of 8 mm diameter and 30 mm length were drilled bilaterally into the proximal area of the tibia and BoneReg-Inject™ or chronOS Inject^®^ were injected into the right leg holes. Comparison of resorption and bone formation in vivo was made by X-ray micro-CT and histological evaluation after a live phase of 12 weeks. Long-term effects of BoneReg-Inject™ were studied using a 13-month live period. Significant differences were observed in (1) amount of new bone within implant (*p* < 0.001), higher in BoneReg-Inject^TM^, (2) signs of cartilage tissue (*p* = 0.003), more pronounced in BoneReg-Inject^TM^, and (3) signs of fibrous tissue (*p* < 0.001), less pronounced in BoneReg-Inject^TM^. Mineral content at 13 months postoperative was significantly higher than at 12 weeks (*p* < 0.001 and *p* < 0.05, for implant core and rim, respectively). The data demonstrate the potential of deacetylated chitin derivatives to stimulate bone formation.

## 1. Introduction

Whenever disease or trauma causes skeletal void or when healing of a fracture is impaired, a common surgical technique is to harvest bone from the iliac crest of the patient and insert it to the injury site to fill the void (autograft). Autografts are regarded as the golden standard in orthopedic surgery. Still, this procedure has a severe drawback, i.e., donor site morbidity such as pain and numbness [1]. Over the last decade, several new products have been introduced, intended to replace bone autografts for filling of skeletal voids and/or for stimulation of bone healing [2,3]. This rapidly growing group of materials is commonly referred to as “synthetic bone graft substitutes”. Despite an intensive search for replacement therapies, no single material has been developed with proven properties that would make it suitable for filling skeletal defects as well as for induction or promotion of bone healing [4]. Instead, two major groups of synthetic bone graft materials can be identified where one group has properties intended to provide bone induction, while in the other group materials have properties that make them suitable to fill bone voids caused by trauma or disease. This second group is by far the larger of the two in terms of product excess, while the group intended for bone induction only consists of a very small number of products. As an example of a material intended for bone induction, synthetic grafts containing bone morphogenic protein (BMP), a recombinant growth factor, are effective in many applications [5]. However they are only approved for a limited number of indications due to safety concerns, such as heterotopic ossification [6,7].

Chitosan is a natural biopolymer that has been shown to promote both differentiation of stem cells into bone-forming osteoblasts and growth of bone colonies in in vitro studies [8,9,10,11], suggesting osteogenic properties. Results of in vivo studies indicate that chitosan alone is sufficient to stimulate osteogenesis [10,12,13]. Studies where other materials with osteogenic, osteo-inductive and/or osteoconductive properties were added to chitosan-based composites to further stimulate the bone healing process have also been described in the literature [14,15].

Chitosan is biodegradable and biocompatible and consists of a linear polysaccharide chain comprising randomly distributed β-(1→4)-linked D-glucosamine and N-acetyl-D-glucosamine units. It is derived from chitin, an insoluble linear β-(1→4)-linked N-acetyl-D-glucosamine polysaccharide, by deacetylation. An increased degree of deacetylation increases the solubility of the polymer by exposing positively charged amine groups of the glucosamine units [16]. Chitin itself serves as a structural material in the exoskeleton of arthropods and in the cell walls of fungi and is the second most abundant biopolymer in nature after cellulose.

In a previous study in a rat model the quantitative assessment of osteogenic effect of chitin derivatives was established and used to compare the osteogenic effect of deacetylated chitin derivatives, with degree of deacetylation from 50–96% [17]. The study showed that injectable implants containing 50% and 70% deacetylated highly pure chitin derivatives gave the highest bone volume regeneration of the materials tested. Therefore it was decided to compare the efficacy of a developmental product referred to as BoneReg-Inject™, which contains Chitobiomer^TM^, and an existing, similar and commercially available product, chronOS Inject^®^, by DePuy Synthes Inc., which served as a predicate device.

BoneReg-Inject™ is a calcium phosphate-based composition comprising tetra-calcium phosphate (TTCP, Ca_4_(PO_4_)_2_O) and α-tricalcium phosphate (α-TCP, α-Ca_3_(PO_4_)_2_) which reacts in situ to form hydroxyapatite (Ca_10_(PO_4_)_6_(OH)_2_) without excess heat formation. Calcium phosphate based bone cement was chosen as a base for the implants because of its widespread clinical use as well as biocompatible and osteoconductive properties [18]. ChronOS Inject^®^, however, is based on β-tricalcium phosphate (β-TCP, β-Ca_3_(PO_4_)_2_), monocalcium phosphate monohydrate (MCPM, Ca(H_2_PO_4_)_2_·H_2_O) and magnesium phosphate (MgHPO_4_·H_2_O), forming brushite (CaHPO_4_·2H_2_O) instead of hydroxyapatite. Both products contain acetylated biopolymers; chronOS Inject^®^ contains sodium hyaluronate and BoneReg-Inject™ contains partially deacetylated chitin.

The aim of the study was to compare the performance of BoneReg-Inject™ and chronOS Inject^®^ in a sheep tibia model with respect to new bone formation, calcification and effect on surrounding tissues on the basis of findings from X-ray micro-CT combined with histology after an observation period of 12 weeks, and to study the long-term effect of the BoneReg-Inject™ implant in a similar model with an observation period of 13 months.

## 2. Materials and Methods

### 2.1. Implants

The chitosan polymer used was produced from chitin from the shells of northern Atlantic shrimps (Pandalus borealis). Deacetylation and purification in multiple steps, involving a series of filtration and precipitation steps, was carried out as described earlier [19]. Extensive analytical work was performed to confirm purity and physico-chemical properties of the material [19,20].

Kits of the BoneReg-Inject™ implants were produced and packaged in the laboratories of Genis hf. The implants consisted of 3.0% *w*/*v* partially deacetylated chitin (chitosan polymer, 50% DDA) (Genis hf., Siglufjörður, Iceland), 30.9% *w*/*v* TTCP/α-TCP (Himed, Old Bethpage, NY, USA), 4.2% *w*/*v* sodium glycerol phosphate (Merck, Darmstadt, Germany), 1.9% *w*/*v* calcium hydroxide (Sigma-Aldrich, St. Louis, MO, USA), 7.7% *w*/*v* phosphoric acid (Merck) and water. These ingredients were packed into a solid and a liquid fraction and sterilized by 20 kGy γ-irradiation. Upon application, solid and liquid components were mixed directly by hand for 3 min; the cement was ready to use 7 min after mixing. The cement was designed to set within 30 min at room temperature but the setting can be delayed up to 2 h by cooling at 2–4 °C. The implant material is soft and injectable, and develops into a non-loadbearing solid (unpublished information) within 24 h, with increasing hydroxyapatite content in the further course of the setting reaction.

The predicate device, ChronOS Inject^®^ (product number 710.066S, Lot No, 2538153), was purchased from Synthes AB, Korta Gatan 9, 171 54 Solna, Sweden.

### 2.2. Experimental Design

It was decided to use a sheep model and to drill holes (8 mm diameter × 30 mm length) into the proximal epiphyseal area of the tibia to inject a standardized volume of the BoneReg-Inject™ implant or chronOS Inject^®^ into the drill holes in order to compare efficacy, resorption, osteoconductive and bone formation (osteogenic) properties in vivo by these two different injectable implants. One hole was drilled into the right leg and one hole was drilled into the left leg of each sheep. Implants were injected into right leg holes. Left leg holes were left empty to serve as control. This model was intended to serve as a representation of a trabecular bone loss associated with bicondylar fracture of the tibial plateau, involving central impaction and causing substantial loss of trabecular bone. The dimensions of the drill holes were chosen to serve this purpose and at the same time to avoid fracture risk [21,22]. The resulting epiphyseal bone void is typically filled with a bone graft or a bone graft substitute. An important aspect of this model is the involvement of cortical and trabecular bone tissues as well as fat and marrow tissues. The comparison was made by applying X-ray micro-CT and histological evaluation of preserved bone tissue after a live phase of 12 weeks (3 months). During a 13 month live period, the long-term effects of BoneReg-Inject™ were tested using the same model. Long term effects of chronOS Inject^®^ were not assessed in this study which represents a limitation.

For the purpose of the study, 45 healthy female sheep, 5–7 years old and of a pristine Nordic breed, were acquired. Thirty sheep were randomly selected for the comparative 3 months study (15 for BoneReg-Inject™ and 15 for chronOS Inject*^®^*) and 15 sheep were randomly selected for the long-term 13 months study on BoneReg-Inject™. The randomization procedure took place before the animals arrived at the surgical facilities, and without the team having seen the sheep. Each sheep already had an identification number. Computer generated random numbers were used to divide the sheep into the 3 test groups, maintaining similar age distribution in all groups. One sheep in the 3 months chronOS Inject^®^ group was diagnosed with *Mycobacterium paratuberculosis* infection in the lower intestines and was euthanized 3 weeks postoperatively. The average age of all animals was 5.9 years; 5.9 years in the BoneReg-Inject*™* short term group, 5.9 years in the chronOS Inject*^®^* group and 5.8 years in the long term BoneReg-Inject™ group. Bodyweight of the animals ranged from 53–86 kg, with an average of 65.6 kg. Average body weight of animals in the BoneReg-Inject™ and chronOS Inject*^®^* group was 64.8 kg and 66.4 kg respectively. The average body weight of animals in the long term BoneReg-Inject^TM^ study was 60.5 kg. Changes in average body weight during the 13 months experiment was +3.24 kg in the BoneReg-Inject™ group, indicating that the animals were in good condition. The changes in body weight in the 3 months comparative study are not available. All 15 sheep in the 3 months BoneReg-Inject™ group, 14 sheep in the 3 months chronOS Inject^®^ group and 15 sheep in the 13 months long term BoneReg-Inject™ group completed the trial alive and without any apparent morbidity.

Before the experiment, sheep were acclimatized for four weeks in the experimental facility (Institute for Experimental Pathology, University of Iceland, Keldur, 112 Reykjavík). During acclimatization and after the operation, animals were kept 3–4 together in a pen. Before the operation, the general health of the animals was confirmed and individual animals assigned to test groups to maintain similar age distribution within the groups. Before the operation the animals were not fed in the afternoon the day before operation and in the morning of the day of operation, but were allowed free access to water. Postoperatively, the sheep were fed hay twice daily (1–2 kg/day) and water ad-libitum.

All experiments were designed and conducted according to the FELASA guidelines. The study protocol was approved by the Icelandic Committee on Animal Experiments with the identification number 0709–0405/2009.

### 2.3. Surgical Procedure and Sample Preparation

At arrival to the operation theater, the sheep were weighed and injected i.v. with Chanazin 10% and Atropine sulfate. Further medication administered during the procedure is listed in Table 1. The sheep were prepared for surgery by shaving both hind upper legs around the knee and the left side of the neck, to expose the jugular vein. An i.v. catheter was inserted in the jugular vein and a bolus of ketamin 100 mg/50 kg administered. Following that a 3–5 mg/kg bolus of propofol was given. When the animal was anesthetized it was positioned on the right side on the operating table. The sheep breathed spontaneously during the operation receiving 6 L 100% oxygen via mask at the nostrils. Every 5–10 min during the operation a bolus of 1–2 mg/kg propofol was given i.v., more often if there were signs of movement. Continous monitoring of the anaesthesia depth was performed by an assigned person with no other duties during the operation. Monitoring of pulse and oxygenation was carried out with pulse oximetry at the tail after shaving it. The right hind leg was fixed to the table and the left leg fixed in an upright position.

The shaved areas on both hind legs were disinfected with Hibiscrub^®^ and subsequently Betadine^®^ solution. The bone surface was accessed through an incision parallel to and over the posterior border of the tibia. The entrance hole was localized centrally between the anterior and posterior borders of the bone at the level of the tuberositas. A hole of 8 mm in diameter was drilled at a right angle through the cortex whereafter the drill was redirected into a 45° upwards direction ending beneath the floor of the tibia plateau. After thorough rinsing of bone debris by flushing with 40–50 mL of sterile, saline water combined with suction, the right leg hole was filled with a standardized amount (1.5 mL) of the experimental test formulation (BoneReg-Inject™ or chronOS Inject^®^). The drill hole in the left leg was left void for comparison. Prior to application the BoneReg-Inject™ product was mixed according to a procedure described in the “Implants” section above and kept at 2–4 °C until application to delay the setting time. ChronOS Inject^®^ comes with a mixing device which was used to mix the two phases, the liquid and the solid, according to instructions from the manufacturer provided with the product. Within the time frame (3 min) given in product instructions the material was injected to fill the bone void. These materials were injected from a 5 mL sterile syringe mounted with a sterile pipette tip, ensuring that the material would fill the entire space of the drill hole from the tibia plateau down, and outwards, to the opening in the cortex. Thereafter the surgical wounds were closed with 4–0 running Vicryl subcutaneous sutures and the skin with 4–0 Ethilon continuous intracutaneous suture.

After surgery and wake-up, the animals were moved back to the sheep pen for recovery and vital signs monitored until they were ambulatory. All animals survived the anaesthesia and operation without complications.

Postoperatively, the animals received medication according to protocol (Table 1). They were monitored daily concerning behaviour, appearance, and appetite as an expression of general health and well-being. No stabilization such as cast was used and the sheep moved around and loaded their limbs as in normal conditions. In general, a drill hole of this size does not cause instability to the bone or the joint as has been confirmed in the literature [23]. Thus, monitoring through X-ray imaging was not considered necessary. The surgical wounds were regularly evaluated with respect to signs of infection, inflammation and dehiscence.

All animals completing the study appeared healthy and behaved normally throughout the study period. One sheep from the chronOS Inject^®^ group was diagnosed 3 weeks postoperatively with *Mycobacterium paratuberculosis* and was immediately euthanized.

At termination after 3 and 13 months of recovery (short and long term study, respectively), sheep were euthanized by 10 mL/50 kg body weight i.v. injection of T 61. Left and right tibia were explanted at the knee joint and carefully cleaned from soft tissues. The tibia was sawed transversally about 4–5 cm below the proximal end maintaining the entire drill hole within the sample. Samples were placed in plastic jars with a tight lid for fixation in a solution of 3.7% formaldehyde in 50 mM phosphate buffer at pH 7.0. During the period of 11–32 days in the fixation media, samples were scanned in a micro CT-scanner. That is, samples had been between 11 and 32 days in the fixation media when CT-scanning was performed. The sample that had experienced the shortest fixation period had been fixed for 11 days, and the sample that had experienced the longest fixation period had been fixed for 32 days at the timepoint of CT-scanning. After the bones had been kept in the fixation media for 21–35 days and scanning was successfully completed, bone samples were sawed transversely through the epiphysis into 3–4 mm slices with a band saw. That is, the sawing of samples was started at day 21 after initial immersion in fixation media and the sawing was finished at day 35 after initial immersion in fixation media. The slices were photographed and returned into the fixation media and kept there at 22–24 °C under occasional stirring until a total fixation time of 73–94 days from initial immersion into the media. Thereafter, the slices were decalcified in 15% EDTA solution at neutral pH for 13 weeks, with 3 changes of solution during the decalcification period, as explained in the following. Selected sections were further prepared for histological evaluation.

### 2.4. X-ray Micro CT Analysis

Scanning was performed in an X-ray micro computed tomography (CT)-scanner (nanotom S, General Electric Inspection Technologies, Wunstorf, Germany). The tibia samples were scanned during the period of 11–32 days postmortem. Samples were fixed in a closed plastic cylinder filled with buffered formalin (3.7% formaldehyde in water), which could be mounted on the rotational table in the CT-scanner. Micro CT scanning was performed by transmitting adjusted dosages of X-rays through the sample while the sample was step wise rotated 360° in intervals of 0.5°, collecting 720 2D images from each sample. Magnification was 2.5, voxel size 20 µm/voxel edge, with exposure time of 3000 ms, frame averaging of 3, and 1 frame skipped. X-ray settings were 110 kV and 120 µA, using tube mode 0 and no filter. Volume reconstruction was performed using the Datos-x software accompanying the CT-scanner. Data analysis of X-ray micro CT data was performed using Volume Graphics Studio Max 2.0 from Volume Graphics GmbH, Heidelberg, Germany.

Mineral content was assessed with the help of a hydroxyapatite (HA) phantom from QRM GmbH, Möhrendorf, Germany, which was scanned together with each sample. The phantom has 5 cylindrical inserts of 5 mm diameter with different partial densities of calcium hydroxyapatite of approximately 0, 50, 200, 800 and 1000 mg HA/cm^3^. The reconstructed CT-data set from each sample was calibrated according to the HA200 insert, which has a partial HA density of 200.3 mg/cm^3^ ± 0.5%. In the following quantitative analysis the HA200 density was chosen as a lower limit, as this was found to include all traces of implanted fillers as well as mineralized tissue. The grayscale value obtained from the calibration for each sample was used to separate the grayscale range into two corresponding density ranges, one of lower density and the other of higher density than the HA200 standard. This value marked the border between mineralized and unmineralized material in the sample and was termed “iso-surface”. The volume of all voxels within the defined volume under analysis (sphere or cylinder), having a grayscale value equal to or greater than the iso-surface value, were added up and defined as the “mineral phase volume” within the total volume under analysis. Hence, in the numerical mineral content calculations, the volume of all voxels with a density equal to HA200 or higher, divided by the total volume being analyzed, was defined as “mineral volume ratio” (volume with a density of ≥200 mg/cm^3^ divided by the total volume).

**Figure 1:**
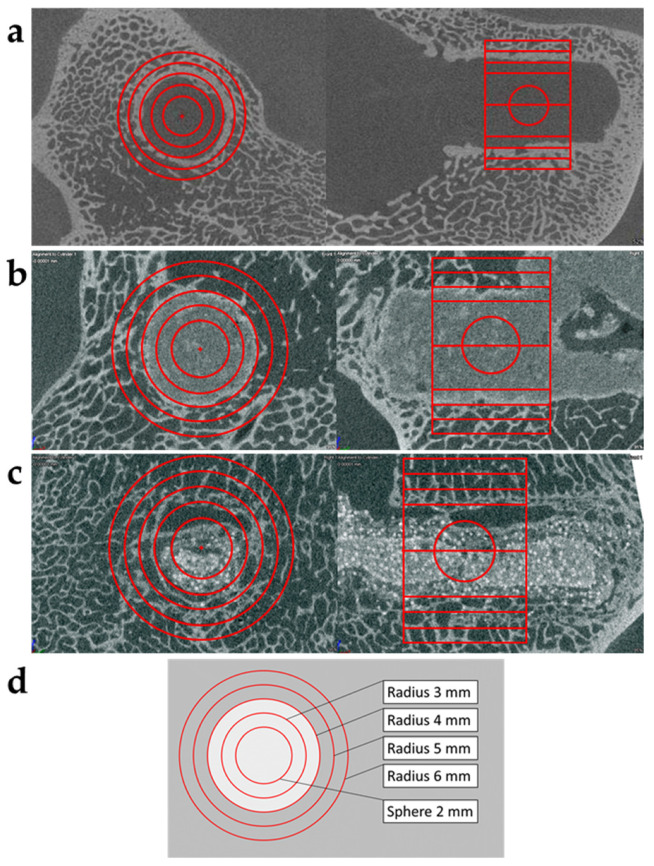
(**a**–**c**) show micro CT constructs in two different planes of samples after 3 months in vivo. (**a**) Sample derived from the left tibia (negative control, empty hole) of an animal receiving BoneReg-Inject™ in right leg drill hole, still essentially void of mineralized tissue. A rim of increased density integrated with the adjacent trabeculum on the edge surrounding the drill hole. The figure shows how virtual cylinders were created, concentric to the drill hole and with different radiuses. (**b**) BoneReg-Inject™ treated sample from the right tibia. A rim of mineralized tissue, well integrated with the adjacent trabecular bone, surrounding the implant, islands of dense material are scattered within the body of the implant. (**c**) chronOS Inject^®^ treated sample from the right tibia. High density granules, characteristic for chronOS Inject^®^ are apparent in the images. Mineralized new tissue is less pronounced in the chronOS Inject^®^ group compared to the BoneReg-Inject™ group. (**d**) Illustration of virtual cylinders created. Length of all cylinders was 8 mm. The largest cylinder has a radius of 6 mm, the smallest has a radius of 3 mm and in the center is a sphere with radius of 2 mm. The cylinder with radius of 4 mm (8 mm in diameter) shows the border of the drill hole.

Mineralization was compared in specific volumes defined by virtual cylinders. A cylinder of 8 mm in length and with a radius of 3 mm was defined and carefully and concentrically orientated with the longitudinal axis in the direction of the drill hole (Figure 1). While keeping the orientation of the cylinder fixed, the radius was increased stepwise to 4, 5 and 6 mm and the mineral phase volume in each cylinder measured. This analysis was repeated in all samples, both empty holes and holes with implant (Figure 1). By subtracting the narrower cylinder from the broader, such as radius 3 mm from radius 4 mm (R4-R3), R5-R4 and R6-R5, mineral phase volume of a 1 mm outer shell (tube) of each cylinder was obtained. Mineral phase volume of the shell of all cylinders was normalized to a standard volume of 1 mm^3^, obtaining mineral content with a density higher than that of the hydroxyapatite standard used.

To estimate mineral density inside the implants a virtual sphere with a radius of 2 mm was defined in the center of all implants/drill holes (Figure 1). Mineral phase volume (volume of voxels with a density ≥ 200 mg/cm^3^) inside the sphere was calculated. Mineral volume ratio was calculated as mineral phase volume/volume of sphere (33.5 mm^3^; r = 2 mm). For comparison to initial density of the two implant types (chronOS Inject^®^ and BoneReg-Inject™), samples of implant composites were allowed to harden ex vivo and mineral phase volume measured in triplicates in 2–3 separate spheres in each sample. Mineral phase volume was measured after 24 h of setting ex vivo at 37 °C in saline water and after 18 months of storage in buffered formaldehyde solution at room temperature in order to exclude storage-influenced changes in mineral phase volume.

### 2.5. Histology

Histological analysis was used for qualitative identification of areas showing dense structures in the micro CT data and to characterize tissue responses to the implant. A ranking system was applied in order to extract statistical data form the histological images.

The medial condyle was sawed off in the sagittal plane, thus forming a plan to lay the tibia on to continue sectioning. Then the tibia plateau was axially (transverse) sawed off (2–3 mm) and subsequently 6–8 axial slices (3–4 mm) were made as far as beyond the cortical entrance of the drill hole. Thereafter all slices were put back into the fixative, carefully ensuring good access of the formaldehyde solution to the entire surface of each slice.

For further histological preparation, 3 slices were selected from each sample. The most proximal slice was the first slice under the tibial plateau where the implant could be seen macroscopically, containing the greatest amount of trabecular bone surrounding the implant. The most distal slice contained the cortical entrance of the drill hole. The third slice was selected from the slices between the proximal and the distal slices with marrow or fat tissue surrounding the implant. The three slices from each tibia were decalcified in 15% EDTA (*w*/*v*) at pH 7.2–8.0 (EDTA (ethylenediaminetetraacetic acid disodium salt dihydrate), Sigma-Aldrich, St. Louis, MO, USA). The process of decalcification was monitored by micro CT analysis. Decalcification period was about 13 weeks, EDTA-solution was renewed every 3–4 weeks. After complete decalcification, the specimens were dehydrated and embedded in paraffin according to conventional histological protocol. To that end, tissue specimens were put into cassettes and tissue rinsed in running tap water for 30 min. The next step was the conventional method of tissue processing overnight in an automatic tissue processor (Tissue Tek VIP, Sakura Finetek, Inc., Torrance, CA, USA) ending with paraffin embedding where the tissue is embedded in paraffin wax at 63 °C. Paraffin blocks were then moved over to a cooling plate for hardening and after that to a microtome for sectioning (Leica RM2255, Leica Microsystems, Wetzlar, Germany). Disposable blades were used (Accu-Edge Disposable Blades, Sakura) for cutting sections at 3 µm thickness. Sections were mounted on coated microscope slides (Star Frost 76 mm× 26 mm, Knittel Glas GmbH, Braunschweig, Germany), dried in a 60 °C oven for 60 min. Ten sections were taken from each paraffin block at 12 µm intervals each and mounted on 10 Star Frost slides marked with specimen number and Roman numerals from I to X. After drying, H&E staining was performed using Shandon instant Hematoxylin and Eosin solution (Sigma-Aldrich).

For histological ranking samples from 13 sheep from the BoneReg-Inject™ group and samples from 12 sheep from the chronOS Inject^®^ group were used, i.e., 25 sheep in total. Three sections per sample were considered. Two observers blindly examined all samples (25 × 3) separately, a total of 25 × 3 × 2 = 150 observations. This gives 6 observations for each sheep. An average was calculated for each sheep. The average was rounded up to the nearest integer of 0, 1, or 2 and the data analyzed for non-parametrical tests. The ranking (0 = none, 1 = moderate presence or 2 = strong presence) was performed with respect to a bone in implant, signs of cartilage tissue and signs of fibrous tissue.

### 2.6. Statistical Analysis

For statistical analysis, SigmaPlot 13 (Systat Software, Inc., San Jose, CA, USA) was used. ANOVA and Student’s *t*-test (two-tailed) were used for parametrical comparison of groups. For non-parametrical comparison, ANOVA on ranks and Dunn’s or Mann-Whitney test was applied. The level of significance was accepted at *p* < 0.05.

## 3. Results

### 3.1. Short Term Study, 3 Months In Vivo

#### 3.1.1. Comparison of Mineral Phase Volume inside BoneReg-Inject^TM^ and ChronOS Inject^®^ Implants

**Figure 2:**
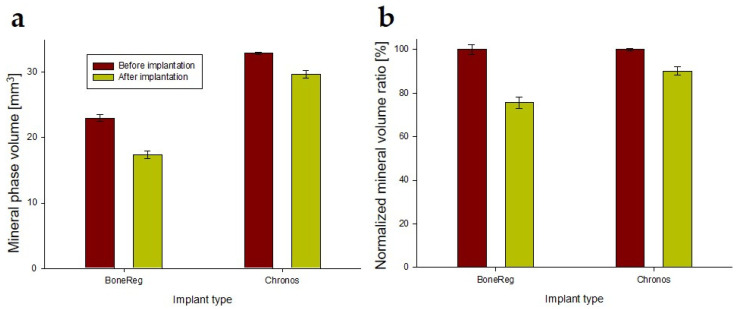
(**a**) Mineral phase volume inside the composites ex vivo (before implantation) and after 3 months in vivo, measured in spheres (radius 2 mm). (**b**) Bars show mineral phase volume before implantation normalized to 100% for both composites and their relative reduction during 3 months in vivo. Mineral phase volume in BoneReg-Inject™ group (*n* = 15) is reduced by 21% and in chronOS Inject^®^ (*n* = 14) by 10%. (Student’s *t*-test; *p* < 0.001). Means and SEM (BoneReg-Inject™ *n* = 15; chronOS Inject^®^ *n* = 14).

Results of mineral phase volume calculations of the 2 mm radius sphere in the center of the implant after 24 h ex vivo and 3 months in vivo, presented in Figure 2a, indicate higher initial mineral phase volume in chronOS Inject^®^ (32.9 mm^3^) compared to BoneReg-Inject™ (23.0 mm^3^). For comparison, the volume of a full sphere is 33.5 mm^3^. However, during 3 months in vivo, the mineral phase volume of chronOS Inject^®^ was reduced to 29.6 mm^3^ while the mineral phase volume of BoneReg-Inject™ was reduced to 18.2 mm^3^. Figure 2b shows the same data when the initial mineral phase volume has been normalized to 100% for both implants. Hence, during 3 months in vivo, the mineral phase volume of BoneReg-Inject™ was reduced by 21% and chronOS Inject^®^ by 10%. These data suggest that in this model, BoneReg-Inject™ breaks down significantly faster than chronOS Inject^®^ (Student’s *t*-test; *p* < 0.001).

#### 3.1.2. Comparison of Mineral Volume Ratio in the Rim of the Implants

**Figure 3:**
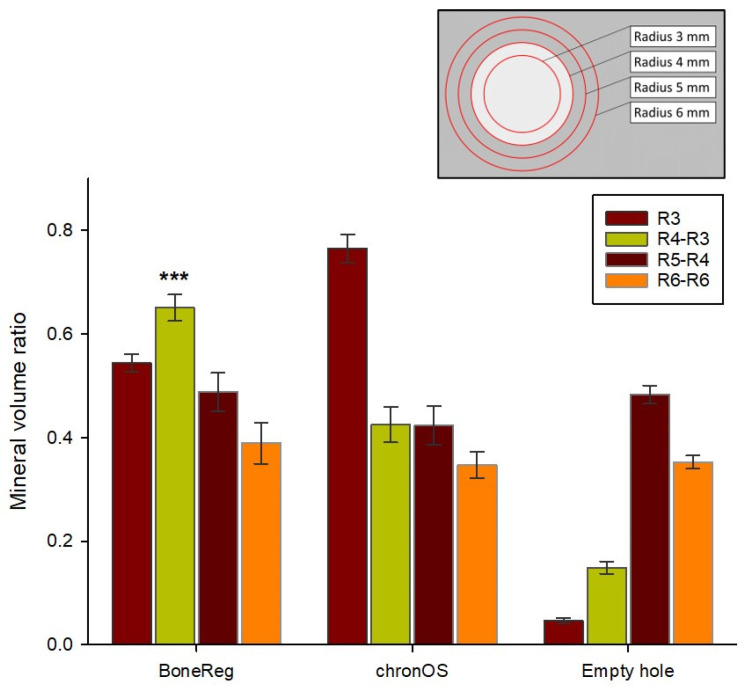
Bars indicate the mean mineral volume ratio in one virtual cylinder and 3 tubes in both experimental groups and the empty holes (negative control). The empty holes in both groups were summed up into a single control group. Results for a cylinder with a 3 mm radius and 3 different tubes with fixed volume are shown; R4-R3, R5-R4, and R6-R5 (see Figure 1 and insert above for location and alignment). The BoneReg-Inject™ group shows a significantly higher mineral volume ratio in a 1 mm rim of the implant (R4-R3) compared to the chronOS Inject^®^ group (Student’s *t*-test *p* < 0.001, indicated by three asterisks in the figure). Values are means and bars are standard error of the mean (SEM). R3 = radius 3 mm, R4 = radius 4 mm, etc. (BoneReg-Inject™ *n* = 15; chronOS Inject^®^ *n* = 14).

Mean mineral volume ratios in concentric virtual cylinders and tubes (1 mm wall thickness) (Figure 1d) represent a gradient of mineral density reaching from the implant and into adjacent tissues (Figure 3). The higher mineral volume ratio of chronOS Inject^®^ in the center of the implants was discussed in the previous section. The same can be observed comparing the mineral volume ratio within cylinders R3. This is reversed for the volume represented by tube R4-R3, forming the rim of the drill hole, where the mineral volume ratio of the BoneReg-Inject™ group was 53% higher than that for chronOS-Inject^®^ group (Student’s *t*-test, *p* < 0.001). On the other hand, no statistical difference was observed between the mineral volume ratio in both materials in the outer cylinders R5-R4 and R6-R5. As expected, the lowest mineral volume ratio was observed in the empty holes (Figure 3). The diameter of the drilling hole is 8 mm (radius 4 mm), suggesting that the BoneReg-Inject™ induced mineralization is a result of bone growth into the drill hole, where the new bone is replacing the implant material as the implant is resolved. A comparison between mineral volume ratio in cylinder R3 on one side and tube R4-R3 on the other side for each type of implant reveals that mineral volume ratio increases on going from R3 to R4-R3 in BoneReg-Inject^TM^, but decreases in chronOS Inject^®^. This points to increased bone formation in the BoneReg-Inject^TM^ material compared to chronOS Inject^®^. Histological examination was used to clarify if the mineralized tissue was new bone, as well as possible differences between the groups in terms of tissue response to the two types of implant.

#### 3.1.3. Comparison of Closure of the Cortical Opening of the Drilled Hole

**Figure 4:**
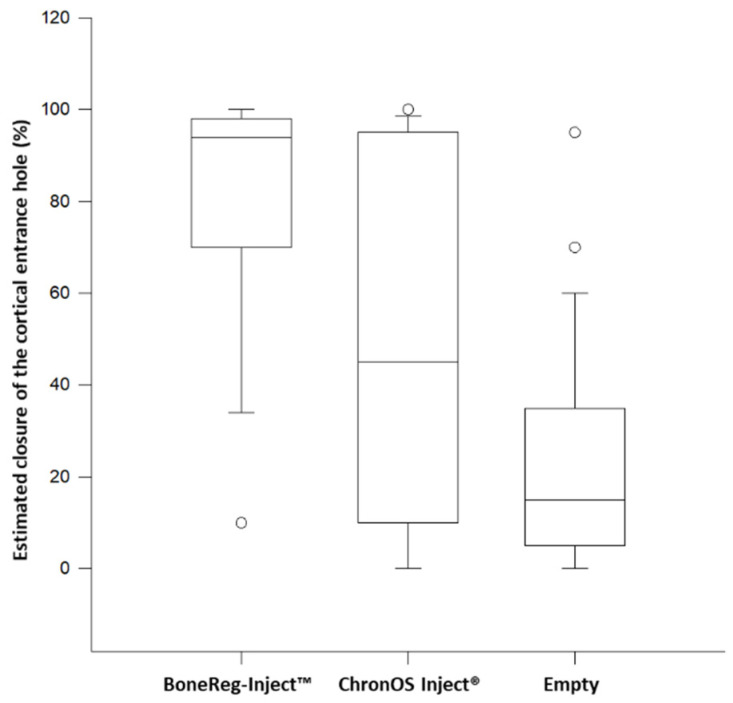
Scoring of percentage closure of cortical entrance hole in the two test groups and the empty holes in both groups 3 months postoperatively shown as box plot based on results from ANOVA on ranks, and only showing a significant difference between BoneReg-Inject™ and empty hole (Empty), Dunn’s Method, *p* < 0.05. Boxes show a 50% confidence limit and bars show a 90% confidence limit. The median is indicated by the horizontal line within the boxes. (BoneReg-Inject™ *n* = 15; chronOS Inject^®^ *n* = 14; Control *n* = 29).

During the 3 months study period, the drill hole through the cortical bone of the tibia was closed to a different degree by bone growth emerging from the edges and striving to fill the hole. Estimated cortical closure of the drilled hole 3 months postoperatively was scored for all samples (0% = no growth, 100% = complete closure of cortical hole). BoneReg-Inject™ group scored highest (94.0%), then chronOS Inject^®^ group (45.0%) and the empty holes had the lowest score (15.0%). However, a statistical difference was revealed between the BoneReg-Inject™ group and the empty hole group (*p* < 0.05), but no difference was found between the ChronOS Inject^®^ and empty hole groups or between the two groups of implant materials (Figure 4).

#### 3.1.4. Histological Examination

**Figure 5:**
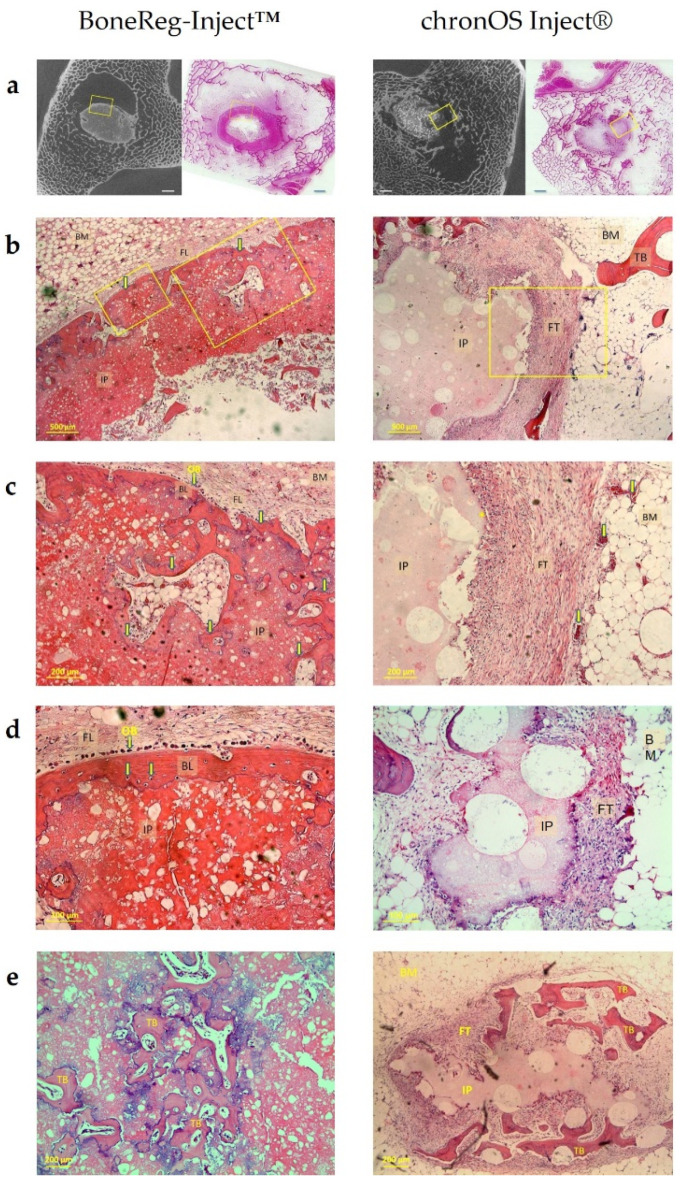
3 months postoperative. (**a**) An alignment of histological sections with two-dimensional cross-sections of micro CT constructs in a bone marrow/trabecular bone area in the right legs of sheep with BoneReg-Inject™ implant (left column) and chronOS Inject^®^ implant (right column). The left images in each column show the two-dimensional micro CT cross sections in the plane aligned with the histological section in the right image. The scale bars are 2 mm. BoneReg-Inject™ column: (**b**) Histological section, a close up of the area of the yellow frame shown. (**a**). The rim of neo-osteoid covering the implant is clearly visible (yellow arrows). BM: Bone marrow, FL: Fibrous layer, IP: Implant. (**c**) A close up of the right yellow frame in (**b**). New bone, connected to the bone shell covering the implant, has grown into the implant (yellow arrows). Characteristic blue deposits seen on the interface between new bone and the implant indicate areas of calcification. Osteoblast-like cells (OB) visible on the outer layer of the new bone shell. (**d**) A close up of the left yellow frame in (**b**). The rim of neo-osteoid covering the implant is evident (BL) and individual osteocytes are visible (yellow arrows). Osteoblast-like cells (OB) are visible on the outer layer of the bone shell. (**e**) Trabecular-like neo-osteoid formation inside the BoneReg-Inject™ implant. Blue indicates regions of on-going calcification. chronOS Inject^®^ column: (**b**) A close up of the yellow frame area shown in (**a**). The fibrous tissue covering the implant is characteristic of the chronOS Inject^®^ group. The characteristic granules are apparent in the implant. FT: Fibrous tissue, TB: Trabecular bone. (**c**) A close up of the yellow framed area in (**b**) showing the implant surrounded by fibrous tissue and bone marrow. It is characteristic for the chronOS Inject^®^ group to see this layer of fibrous tissue surrounding the implant (*). Blood vessels with red blood cells are evident both in the bone marrow and fibrous tissue layer (yellow arrows). (**d**) Implant surrounded by fibrous tissue. (**e**) Trabecular-like neo-osteoid (TB) formed inside the layer of fibrous tissue replacing the chronOS Inject^®^ implant (IP), as it resolves. This formation of new bone was generally rare in the chronOS Inject^®^ group and was poorly integrated with the existing surrounding trabecular bone.

Figure 5 shows a series of images selected from two animals from each group representing a characteristic appearance of histological images from BoneReg-Inject™ and chronOS Inject^®^. Histological images are aligned with the corresponding micro CT sections in order to compare how dense structures in the micro CT data align with newly formed bone tissue. Figure 5e represents samples from each of the treatment groups showing the best case observed of neo-osteoid formation inside the BoneReg-Inject™ implant and the chronOS Inject^®^ implant.

A qualitative pathological evaluation, based on three sections from the right leg of each animal. revealed differences between BoneReg-Inject™ and the predicate device.

BoneReg-Inject™ Group: The implanted material was intensely eosinophilic and finely granular. Around the implant was a rim of neo-osteoid, its thickness approximately that of a normal bony trabecula. In some cases, there were signs of the inner border of the new osteoid undergoing calcification. The outer periphery of the rim was lined by a row of osteoblasts, osteoclasts being virtually absent. In some cases, neo-osteoid at the periphery of the rim was seen to connect with adjacent preexistent bone. No foreign body reaction was noted around the implant.

ChronOS Inject^®^ Group: The implanted material was homogenous, weakly eosinophilic and punctuated by large perfectly round defects. Around the implant, most cases demonstrated signs of marked tissue reaction, consisting of granulation tissue, i.e., newly formed vessels, fibroblasts, and lymphocytes, as well as foreign body reaction, including epithelioid histiocytes and multinucleated giant cells of the foreign body type. No cytoplasmic inclusions were noted in giant cells. Neo-osteoid was inconspicuous, seen only in a minority of cases. Where seen, neo-osteoid was haphazardly present, without an apparent connection to preexistent bone trabeculae.

A histological ranking was used for comparison of samples from both groups. Samples were rated from 0–2 in terms of parameters of interest regarding group differences in the histological images. Significant differences between the two groups were revealed concerning: (1) amount of new bone within the implant (*p* < 0.001), (2) signs of cartilage tissue (*p* = 0.003) and (3) signs of fibrous tissue (*p* < 0.001). Table 2 shows the ranking outcome. The histological ranking showed more bone in implant, more signs of cartilage tissue and less fibrous tissue for BoneReg-Inject^TM^ implants compared to the predicate device.

### 3.2. Long Term Effect of BoneReg-Inject^TM^, 13 Months In Vivo

#### 3.2.1. X-ray Micro CT Analysis

Comparison of micro CT data of BoneReg-Inject™ implants at 3 and 13 months postoperative revealed continuous mineralization during the 10 months period. The new mineralized tissue appeared well integrated with the adjacent trabecular bone and appeared to be increasingly migrating into the implanted material.

**Figure 6:**
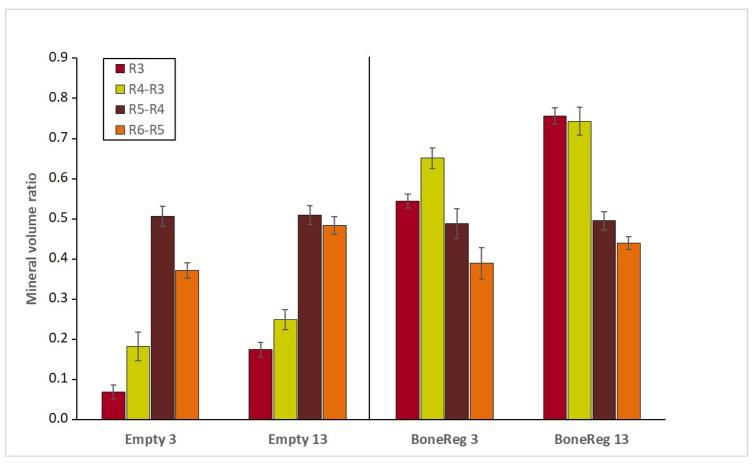
Mineral volume ratio in a virtual cylinder (R3) and tubes R4-R3, R5-R4 and R6-R5 in BoneReg-Inject™ samples after 3 months (BoneReg 3) and 13 months (BoneReg 13) and in the empty control (Empty 3 and Empty 13). Mean and SEM (*n* = 15 for each treatment). Mineral content within the implanted scaffold at 13 months postoperatively was significantly higher than at 3 months postoperatively (R3 *t*-test, *p* < 0.001 and R4-R3 *t*-test, *p* < 0.05). In the empty drill holes, mineral content within R3 was significantly higher after 13 months (*t*-test, *p* < 0.001). A significant increase in mineral content was also observed in the trabecular bone outside the empty holes (R6-R5, *t*-test, *p* < 0.001), potentially reflecting a response to counteract weakening of the bone due to the drill hole.

Mineral volumes obtained using virtual cylinders defining specific volumes inside and surrounding the BoneReg-Inject™ implants were used to compare 3 and 13 months postoperatively (Figure 6).

When comparing the empty holes (Empty 3 and Empty 13), a significant increase is observed in the innermost cylinder (R3) during the 10 month period (*t*-test, *p* < 0.001). No significant difference is observed in the rim of the drill hole (R4-R3 or R5-R4) but there is a significant increase in the trabecular bone density in R6-R5 during the 10 months period (*t*-test, *p* < 0.001). This might reflect a response to a weakening of the tissue structure imposed by the drill hole.

Comparing short and long term effect of BoneReg-Inject™ implants (BoneReg 3 versus BoneReg 13, Figure 6), there is a significant increase in mineral content of the R3 cylinders (*t*-test, *p* < 0.001) as well as for the R4-R3 tubes (*t*-test, *p* < 0.05). The mineral content of the adjacent outer trabecular bone tissue, R5-R4, and R6-R5, is not increased over time. This suggests that the implant possesses both osteoconductive and osteogenic activities, leading to the formation of new mineralized bone tissue in the implanted material, remodeling the implanted scaffold into a new mineralized bone tissue.

#### 3.2.2. Histological Evaluation

**Figure 7:**
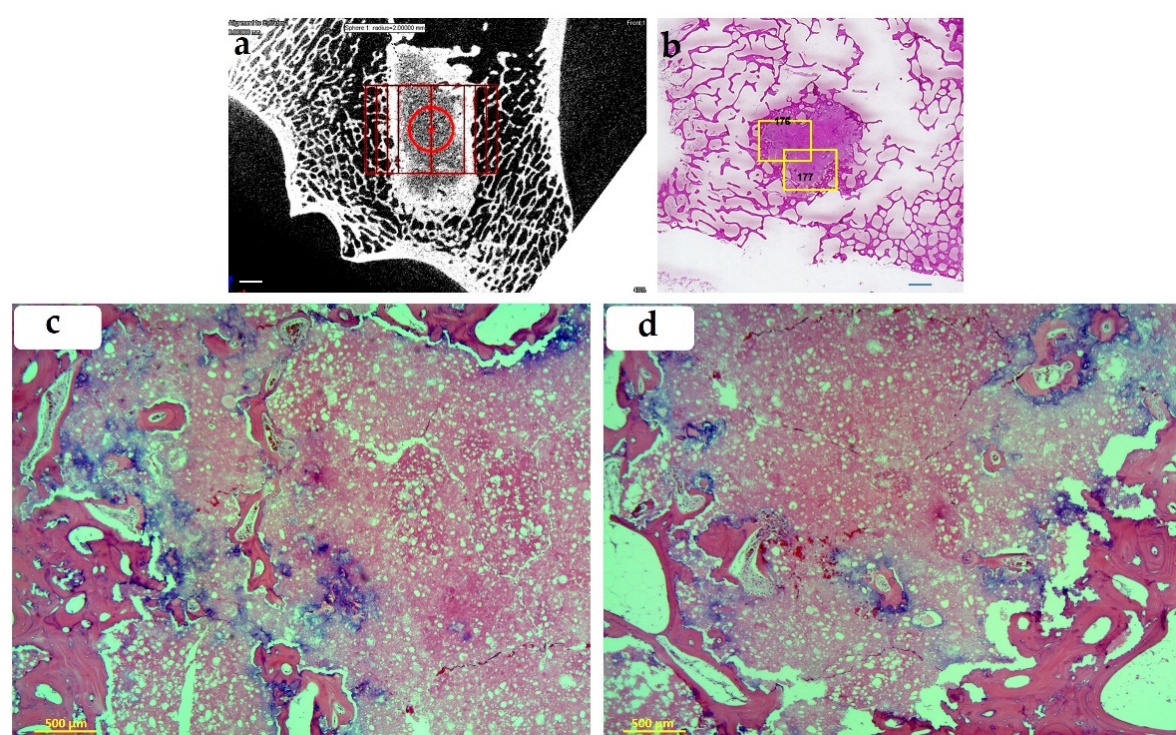
BoneReg-Inject™. (**a**) Micro CT image (scale bar = 2 mm) and (**b**) a histological slide showing BoneReg-Inject™ implant at 13 months postoperative (scale bar = 2 mm). New bone is well integrated with the surrounding trabecular bone. Yellow frames on the histological slide refer to regions in focus in (**c**) (176) and (**d**) (177). In this animal, the micro CT image shows relatively well-developed colonization of mineralized tissue inside the implant. The histological slide indicates trabecular-like neo-osteoid replacing the resolved outer rim of the implant and integrating into the surrounding trabecular bone. (**c**,**d**) show neo-osteoid replacing the resolved surface of the implant and forming islands scattered inside the mass of the implant. Blue indicates calcification. Samples stained with hematoxylin and eosin.

In the long-term 13 month group, the BoneReg-Inject^TM^ implant is eosinophilic and finely granular in appearance. Around the implant is a rim of new bone with a trabecular structure. Trabecular spaces are filled with non-reactive bone marrow. The outer periphery of the new bone is lined by osteoblasts and in some cases connects with adjacent preexistent bone. Bone in-growth is observed in the implanted material, in many cases including its central part. The border between the implant and the new osteoid is often covered with blue deposits indicating calcification. There were no histological signs of inflammation. An example of a series of histological images is shown in Figure 7.

## 4. Discussion

A significant difference was observed between the two experimental groups, BoneReg-Inject™ and chronOS Inject^®^, with more bone regeneration in the BoneReg-Inject™ group.

The BoneReg-Inject™ group was characterized by the formation of a dense mineralized new bone tissue, surrounding the entire implant with scattered islands of new bone formation throughout the interior of the implant.

Quantitative analysis of mineral volume ratio revealed that the BoneReg-Inject™ implant underwent more rapid resolution compared to the chronOS Inject^®^ implant. Higher mineral volume ratio was measured on the periphery of the BoneReg-Inject™ implant pointing to a more pronounced formation of new bone. Histological ranking results supported this finding.

In the long term group implanted with BoneReg-Inject™, a continuing resolution of the implant was apparent and a continuous bone formation within the implant was observed. This indicates that the BoneReg-Inject™ bone void filler comprises a sustained stimulation of bone formation and is replaced by new bone tissue as the implant is resolved with time.

The results obtained here can be viewed in the light of results from experiments that our group performed in rat mandibular bone [17]. It was found that implants containing 50–70% DDA chitosan polymer may stimulate new bone growth. The main increase in bone volume took place in bone tissue surrounding the drill hole in the mandibular bone. The findings suggested that the increase in bone volume was the combined result of biomechanical stresses around the drill hole, and the implant material or components thereof. In the current experiment the same 50% DDA chitosan polymer was used. Here, a significant difference in mineral volume ratio outside the BoneReg-Inject^TM^ implants compared to the chronOS Inject^®^ implants was not observed, but a non-significant trend towards an increased mineral volume ratio outside the BoneReg-Inject^TM^ implants could be identified. Less biomechanical stress is expected around the drill hole in the current study as compared to the rat study, as the ratio between the hole and the surrounding bone is lower, thus displaying relatively lower levels of mechanical stress. This might lead to a lower factor of biomechanical stimulation of bone growth. However, extensive formation of new bone was observed within the implant in the current study. This might reflect the better containment of the implant, so that biomechanical stimulation through the implant is more effective than in the rat mandibular model. Another explanation may be found in the differences in the biological nature of the mandibular bone compared to the tibial bone (intramembranous ossification vs. endochondral ossification) leading to different conditions for the evolvement of the effect of the chitosan polymer and thus bone formation within the implant in the tibia.

Various chitosan composites and chitosan coatings for bone tissue engineering materials have been tested and reported. A common feature of the research is that the chitosan used is highly deacetylated, as opposed to the chitosan employed in the current research. Chitosan preparation and dissolution strategies vary and different composite components are used, all potentially influencing the biological properties and activity of the chitosan. This often makes the comparison between results from different research groups complicated. Some researchers have observed a neutral effect of the use of chitosan in bone implants [24,25]. Several recent papers indicate a positive influence of chitosan on bone regeneration. A chitosan/dicarboxylic acid scaffold was shown in vivo to be an effective bone regeneration material with good osteoinductive and osteoconductive properties [26]. In a review paper, titanium surfaces functionalized with chitosan were concluded to have greater osseointegration capacity than uncoated controls [27]. Thitiset et al. [28], used chitoologosaccharide for preparation of composites that did show promising results in cell culture, as well as for new bone formation in vivo. The results from Thitiset et al. are remarkable in the light of our ideas on the mechanism of action of chitosan in bone regeneration where chitooligosachharides play a central role [17].

## 5. Conclusions

Our overall conclusion is that, in this non-weightbearing sheep tibia model, BoneReg-Inject™ showed osteogenic and osteoconductive properties without identifiable foreign body reaction. In the same model chronOS Inject^®^ provokes foreign body reactions surrounding the implant with significantly less signs of new bone tissue formation. A long-term study indicates that the BoneReg-Inject™ implant stimulates sustained bone formation and is replaced by normal-looking new bone tissue.

For clinical use in bone defects, the current results point to an opportunity for the application of implant materials containing specific, partially deacetylated chitin derivatives, as an active component. The materials stimulate bone formation within the defect over an extended period and, while slowly degrading, facilitate bone ingrowth into the defect.

## Figures and Tables

**Table 1:** Medication and dosages.

Premedication	Chanazin 10% (xylazin) Dosage: 0.1 mL/50 kg Body Weight i.v. and Atropine Sulfate, 1 mL s.c.
General anaesthesia	Narketan Vet. (ketamin 100 mg/mL). Dosage: 1 mL/50 kg i.v. Propofol 10 mg/mL. Bolus at induction 3–5 mg/kg. Maintainance with 1–2 mg/kg every 10 min during the operation and if signs of movement
Local anaesthesia	Xylocain adrenalin 2% and Marcain adrenalin, 5 mg/mL mixed together
Analgesis	Finadyne vet (flunixin) 1 mL/50 kg body weight on day of operation and once a day during the next consecutive 3 days
Antibiotica treatment	Duplocillin LA, 1 mL/10 kg before operation and twice every second day after operation
Euthanasia	T 61, 10 mL/50 kg

**Table 2:** Mann-Whitney rank sum test results. A total comparison of 25 sheep tibia, 13 of BoneReg-Inject™ and 12 of chronOS Inject^®^ implants. When ranking histological samples (0, 1 or 2) with respect to a bone in implant, signs of cartilage tissue and signs of fibrous tissue, a statistical evaluation showed significant differences between the two groups. 0 = none; 1 = moderate presence; 2 = strong presence.

	BoneReg	BoneReg	BoneReg	ChronOS	ChronOS	ChronOS	
	Median	25%	75%	Median	25%	75%	*p*
Bone in implant	1	0	1	0	0	0	<0.001
Cartilage	0	0	1	0	0	0	0.003
Fibrous tissue	0	0	0	1	0.5	2	<0.001

## Data Availability

The data presented in this study are available on request from the corresponding author. The data are not publicly available due to the size of the X-ray microCT datasets.
